# Thyroid Storm Precipitated by Duodenal Ulcer Perforation

**DOI:** 10.1155/2015/750390

**Published:** 2015-03-09

**Authors:** Shoko Natsuda, Yomi Nakashima, Ichiro Horie, Takao Ando, Atsushi Kawakami

**Affiliations:** Department of Endocrinology and Metabolism, Nagasaki University Hospital, Nagasaki 852-8501, Japan

## Abstract

Thyroid storm is a rare and life-threatening complication of thyrotoxicosis that requires prompt treatment. Thyroid storm is also known to be associated with precipitating events. The simultaneous treatment of thyroid storm and its precipitant, when they are recognized, in a patient is recommended; otherwise such disorders, including thyroid storm, can exacerbate each other. Here we report the case of a thyroid storm patient (a 55-year-old Japanese male) complicated with a perforated duodenal ulcer. The patient was successfully treated with intensive treatment for thyroid storm and a prompt operation. Although it is believed that peptic ulcer rarely coexists with hyperthyroidism, among patients with thyroid storm, perforation of a peptic ulcer has been reported as one of the causes of fatal outcome. We determined that surgical intervention was required in this patient, reported despite ongoing severe thyrotoxicosis, and reported herein a successful outcome.

## 1. Introduction

Thyroid storm is a rare complication of thyrotoxicosis, most often associated with Graves' disease. As a life-threatening development, thyroid storm requires prompt treatment. The typical symptoms of thyroid storm are hyperthermia and mental disturbance, along with thyrotoxic symptoms. Thyroid storm is also known to be associated with precipitating events, such as the withdrawal of an antithyroid drug, radioiodine therapy, infection, and surgery including thyroid itself [1]. Distinguishing the components of thyroid storm from manifestations of the underlying precipitant can at times be challenging. For example, unexplained abdominal pain is not an infrequent complaint in patients presenting with thyroid storm [2]. However, once recognized, both thyroid storm and its precipitants require immediate treatment.

Here we report the case of a patient with thyroid storm and a perforated duodenal ulcer. We also discuss the importance of the recognition of a perforation of a peptic ulcer in patients with thyroid storm.

## 2. Case Presentation

A 55-year-old Japanese male was admitted to our hospital in April 2014 because of severe abdominal pain that developed suddenly. He had been diagnosed with Buerger's disease at 50 years of age and required low-dose loxoprofen (~60 mg a day) for his leg pain in the winter. He also had had an interferon treatment for chronic hepatitis C, but his HCV-RNA remained positive. He had never developed peptic ulcer disease before. He had been smoking for 30 yrs.

He had noticed his leg edema a few months earlier and a hand tremor a few weeks prior to his admission. He visited our hospital with complaints of leg edema several days prior to the admission. He was tachycardic (138 beats per min) with a goiter, and there was atrial fibrillation on ECG. He was thyrotoxic (free T3 14.89 pg/mL, free T4 4.11 ng/dL, and TSH < 0.005 *μ*IU/mL) with positive TSH receptor antibodies (15.7 IU/L, reference range < 1.0) and TSH-stimulating antibodies (1143%, reference range < 180%). He was treated with 15 mg/day of methimazole and 30 mg/day of propranolol a week before the admission. He did not notice palpitations or weight loss and there were no complaints of nausea, vomiting, or diarrhea at his first presentation to our hospital.

**Figure 1:**
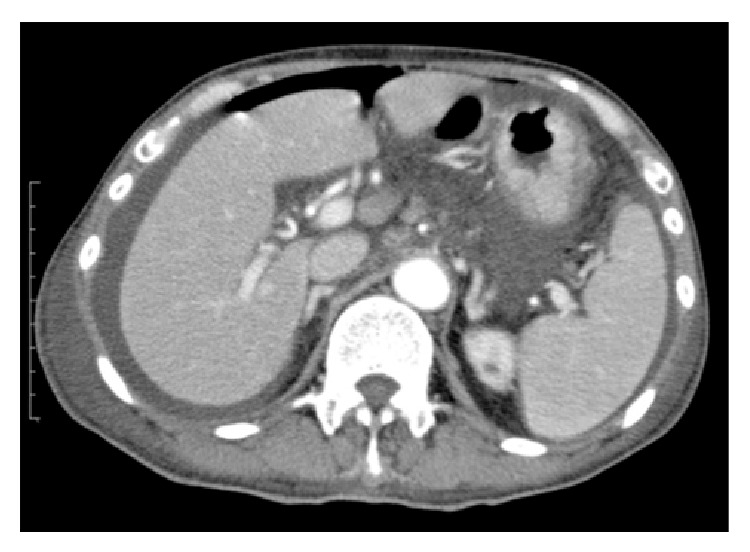
Detection of free air in the patient with thyroid storm, a 55-year-old Japanese male. Contrast-enhanced abdominal CT shows the presence of free air in the surface of the liver associated with ascites.

On admission to our hospital because of acute severe abdominal pain, the patient's consciousness was disturbed (E4V2M6 on the Glasgow coma scale). He was febrile (39.3°C) with irregular heartbeats (121 per min) and normotensive (105/53 mmHg). There was swelling of his abdomen with peritoneal signs of marked abdominal tenderness, rebound tenderness, and percussion pain. These findings suggested peritonitis. The ascites obtained was cloudy yellow exudate with leukocytosis (25 nucleated cells/mm^3^). The patient underwent contrast-enhanced abdominal computed tomography (CT), and there was free air on the surface of the liver along with massive ascites (Figure 1).

The patient's condition suggested thyroid storm triggered by gastrointestinal perforation considering his clinical signs and symptoms with the very short period of treatment with methimazole. His thyroid storm score indeed met the criterion of thyroid storm: 50 points in Bruch-Wartosky-Score [3] and alternate combination of TS2 in the JTA classification [4]. We were not able to determine the thyroid function of the patient because our laboratory does not measure thyroid hormone during the weekends.

Emergency abdominal surgery was performed to treat the patient's gastrointestinal perforation on the day of his admission. This was because the patient's abdominal symptoms were worsening progressively and we considered that the conservative management would not be effective. The patient was prepared with hydrocortisone 250 mg/day. Since oral intake was contraindicated, we did not use methimazole before the operation: intravenous or rectal methimazole was in not available in our hospital. We assumed the patient was given enough iodine from the contrast media used in abdominal CT; it contains 300 mg/mL of iodide and the actual amount used was 100 mL. Landiolol hydrochloride (~0.04 mg/kg/min) was used to control his tachycardia. His pulse rates were 130–160 bpm and his body temperature was 37-38°C during operation. We were able to identify the perforated duodenal ulcer located in the anterior wall of the duodenum, and the lesion was wrapped with the greater omentum. The patient was treated with hydrocortisone (250 mg/day) for a few days after the operation. His thyroid was treated successfully with methimazole (5–10 mg/day) thereafter.

Postoperatively, the patient's case was complicated with a fungal peritoneal infection, and this was successfully treated with an antifungal agent and peritoneal drainage. His thyroid was treated with methimazole and his duodenal ulcer was treated with a proton-pump inhibitor. The patient was negative for anti-*Helicobacter pylori* IgG.

## 3. Discussion

The frequency of peptic ulcer and its perforation is known to be increased by several factors including old age, prior peptic ulcers, gastrointestinal bleeding, infection with* Helicobacter pylori*, and treatment with low-dose aspirin, antiplatelet, corticosteroid, or anticoagulant medications [5]. Our patient was a smoker who was taking 60 mg/day of loxoprofen. Smoking has been shown to be responsible for approximately one-fourth of the incidence of peptic ulcer disease [6]. Similarly, nonsteroidal anti-inflammatory drugs (NSAIDs) have been shown to be responsible for approximately another one-fourth of the incidence of peptic ulcer disease [6], and it was reported that chronic users of NSAIDs may have a five- to eight-times increased risk of developing perforation of a peptic ulcer [7, 8]. It has been shown that the incidence of gastroduodenal ulcers was 27.6% in Japanese volunteers taking 120 mg/day of loxoprofen for 2 weeks but only 2.7% in those taking a placebo [9]. Thus, the present patient's NSAID use and smoking must have played pivotal roles in the development of his perforated duodenal ulcer.

Acute perforation of peptic ulcers has been reported equally in males and females, with the median age of 56 years [10]. It should be noted that the common presenting symptoms were the sudden onset of severe abdominal pain in almost all of the reported patients and fever in more than half of the patients [10]. Patients with a perforated peptic ulcer should be treated intensively, including the treatment of sepsis as a complication when it is observed. The mortality rates reported for surgically treated perforated duodenal ulcers are 4.3%–17.1% [11, 12]. A perforation of a peptic ulcer may seal spontaneously [13], and thus conservative treatment can be considered safer especially in patients with comorbidity; however, it has been reported that delayed surgery (>24 h) was one of the factors of poor clinical outcome [14]. The outcome of these patients was described as predictable by three risk factors: major medical illness, preoperative shock, and long-standing perforation (>24 h) [15]. Thus, patients undergoing conservative medical management due to extensive comorbidities may still require surgical intervention once their condition deteriorate.

It has long been believed that peptic ulcer rarely coexists with hyperthyroidism [16]. This might be due to the difference in the autonomic activity associated with the two disorders: parasympathetic-dominant activity in peptic ulcer disease and sympathetic-dominant activity in hyperthyroidism [17]. It was shown that acid secretion is reduced in patients with hyperthyroidism [18]. In the same context, one study showed no abnormality in acid secretion in patients with hyperthyroidism, but acid secretion was shown to increase upon treatment for hyperthyroidism [19]. These findings might be clues to the mechanisms underlying the rarity of peptic ulcer disease in patients with hyperthyroidism.

Akamizu et al. recently proposed diagnostic criteria for thyroid storm after studying the cases of 356 patients with either definite or suspected thyroid storm, collected by a nationwide survey in Japan [4]. The mortality rate of the thyroid storm patients in the study was 10.7%, similar to other studies [4, 20]. The major causes of death reported by Akamizu et al. were multiple organ failure and congestive heart failure. They included two fatal cases caused by perforation of the gastrointestinal tract (5% of the fatal cases). These two cases were not reported in the English literature, but the perforation of the gastrointestinal tract was initially treated conservatively due to the patient's poor general condition in one of the cases.

In the English literature, the first report of a patient with thyroid storm complicated with perforated peptic ulcer disease was published in 2008 [21]. Another hyperthyroid patient without thyroid storm complicated with perforated peptic ulcer was reported in 2006 [22]. These patients were treated successfully with rigorous medical therapy against hyperthyroidism and with surgical closure of the perforated peptic ulcer. Nonetheless, the oral intake of antithyroid drugs is contraindicated in patients with perforation of the gastrointestinal tract; this would hamper the treatment of the underlying thyroid disorder, potentially leading to an exacerbation of the general condition of a patient.

In conclusion, perforation of the gastrointestinal tract in patients with thyrotoxicosis seems to be extremely rare, but such patients should be treated intensively against underlying thyroid disorders as well as peptic ulcer disease, including the surgical closure of the perforation.
